# 120 Impact of Undergraduate Clinical Research Experience: Highlighting the UCLA Clinical and Translational Science Institute Research Associates Program (CTSI-RAP) – CORRIGENDUM

**DOI:** 10.1017/cts.2024.560

**Published:** 2024-08-29

**Authors:** Amanda Piring, Jim Morrison, Noah Federman, Laurie Shaker-Irwin, Sam Duong-Brett

The above abstract published with an error in the author order. The correct order is shown below.

Amanda Piring, Sam Duong-Brett, Jim Morrison, Noah Federman and Laurie Shaker-Irwin
